# Pathophysiology and management of valvular disease in patients with destination left ventricular assist devices

**DOI:** 10.3389/fcvm.2022.1029825

**Published:** 2022-11-03

**Authors:** Ioannis Dimarakis, Paul Callan, Maziar Khorsandi, Jay D. Pal, Claudio A. Bravo, Claudius Mahr, Jeffrey E. Keenan

**Affiliations:** ^1^Division of Cardiothoracic Surgery, Department of Surgery, University of Washington Medical Center, Seattle, WA, United States; ^2^Department of Cardiothoracic Transplantation, Manchester University Hospital NHS Foundation Trust, Wythenshawe Hospital, Manchester, United Kingdom; ^3^Division of Cardiology, Department of Medicine, University of Washington Medical Center, Seattle, WA, United States

**Keywords:** heart failure, left ventricular assist device, LVAD, tricuspid regurgitation, aortic insufficiency, mitral regurgitation

## Abstract

Over the last two decades, implantable continuous flow left ventricular assist devices (LVAD) have proven to be invaluable tools for the management of selected advanced heart failure patients, improving patient longevity and quality of life. The presence of concomitant valvular pathology, including that involving the tricuspid, mitral, and aortic valve, has important implications relating to the decision to move forward with LVAD implantation. Furthermore, the presence of concomitant valvular pathology often influences the surgical strategy for LVAD implantation. Concomitant valve repair or replacement is not uncommonly required in such circumstances, which increases surgical complexity and has demonstrated prognostic implications both short and longer term following LVAD implantation. Beyond the index operation, it is also well established that certain valvular pathologies may develop or worsen over time following LVAD support. The presence of pre-existing valvular pathology or that which develops following LVAD implant is of particular importance to the destination therapy LVAD patient population. As these patients are not expected to have the opportunity for heart transplantation in the future, optimization of LVAD support including ameliorating valvular disease is critical for the maximization of patient longevity and quality of life. As collective experience has grown over time, the ability of clinicians to effectively address concomitant valvular pathology in LVAD patients has improved in the pre-implant, implant, and post-implant phase, through both medical management and procedural optimization. Nevertheless, there remains uncertainty over many facets of concomitant valvular pathology in advanced heart failure patients, and the understanding of how to best approach these conditions in the LVAD patient population continues to evolve. Herein, we present a comprehensive review of the current state of the field relating to the pathophysiology and management of valvular disease in destination LVAD patients.

## Introduction

Albeit the term “destination” appeared in the literature in the mid-nineties (1), destination therapy (DT) in reference to the implantation of durable mechanical support devices for advanced heart failure became embedded in the heart failure lexicon with the publication of the Randomized Evaluation of Mechanical Assistance for the Treatment of Congestive Heart Failure (REMATCH) study in 2001 (2). In this multicenter prospective randomized trial, 128 end-stage heart failure patients ineligible for heart transplantation were randomized to either left ventricular assist device (LVAD) with the first-generation HeartMate VE (Thoratec Corporation, Pleasanton, CA) or to receive optimal medical management. Investigators reported a 48% reduction in the risk of death from any cause in the LVAD group as compared with the medical-therapy group, with Kaplan–Meier estimates of survival at 1 and 2 years being 52 vs. 25%, and 23 vs. 8%, respectively. In November 2002 the Food and Drug Administration (FDA) expanded the approved indications for the HeartMate™ SNAP VE LVAS device from bridge to transplantation to include DT; the approval order stated that the device “is now also indicated for use in patients with New York Heart Association Class IV end-stage left ventricular failure who have received optimal medical therapy for at least 60 of the last 90 days, who have a life expectancy of <2 years, and who are not eligible for cardiac transplantation” (3).

Since this initial FDA approval of implantable LVAD for DT, LVAD technology and strategies for managing LVAD patients have evolved considerably. Pre-existing native valvular heart disease as well as in-situ valvular prostheses were traditionally considered a contraindication to LVAD implantation (4). Due to the rapid initial expansion seen in the field of mechanical circulatory support, it was shown early on that surgical intervention could be undertaken to facilitate LVAD candidacy in patients with pre-existing valvular pathology with acceptable early morbidity or mortality (4, 5). The International Society for Heart and Lung Transplantation (ISHLT) in 2013 issued a list of recommendations providing guidance to all aspects of clinical management including associated valvular heart disease; an evidence-based approach was followed with majority of recommendations being level of evidence C or consensus agreement (6). Since then, the literature has been enriched by numerous clinical studies providing further insight into the underlying pathophysiology and associated mid- and long-term clinical outcomes.

In this review, we critically appraise the impact of valvular heart disease on LVAD patient outcomes and delineate the current state of the field regarding how concomitant valve disease is addressed both medically and surgically in this population. Furthermore, we review current concepts of development of de-novo valvular pathology post LVAD implantation and proposed preventative strategies.

## Aortic valve

In a conventional arrangement, continuous-flow LVADs funnel left ventricular blood into the ascending aorta creating a transvalvular pressure gradient across the aortic valve. Theoretically, when the gradient is >0, the aortic valve remains persistently closed throughout the cardiac cycle altering not only physiological flow patterns within the aortic root, but also the distribution of mechanical stress on the proximal ascending aorta and aortic valve apparatus. The ensuing pathophysiological changes of leaflet deterioration, commissural fusion, and aortic sinus dilation may lead to worsening of pre-existing aortic insufficiency (AI) or lead to the development of *de novo* AI.

AI in the context of LVAD physiology effectively creates a closed-loop circulation between the ascending aorta and left ventricle, leading to suboptimal left ventricular unloading, reduced peripheral perfusion, and eventually recurrence of heart failure symptoms. Multiple studies have documented an increasing incidence of AI with the introduction of second-generation LVADs (7, 8). Furthermore, in a systematic review and meta-analysis of *de novo* AI during log-term LVAD support, investigators reported a pooled incidence of significant AI of 25% (11%−42%) during a support period of 412 ± 281 days (9). ISHLT guidelines have recommended consideration for surgical intervention during device implantation in cases with more than mild aortic insufficiency (Class 1, Level of evidence 3) (6). A comprehensive list of representative studies regarding the interplay of AI during LVAD implantation is provided in Table 1.

**Table 1:** Key studies in chronological order of publication reporting on the interaction of AI and LVAD implantation.

**References**	**Design**	**Year**	**Study groups**	***N***	**Main outcome**
Cowger et al. (7)	Retrospective Single institutional	2010	LVAD (HM-XVE/HM II)	78	Early evidence of progressive nature of AI post LVAD implantation
Pak et al. (8)	Retrospective Single institutional	2010	LVAD (HM-XVE/HM II)	130	*De novo* AI with LVADs shown to occur frequently
Toda et al. (10)	Retrospective Single institutional	2011	LVAD (Toyobo-VAS/HM II/Novacor)	47	Significantly worse survival in patients who developed *de novo* AI at 1 year after LVAD implantation
Dranishnikov et al.	Retrospective	2012	LVAD (HVAD/HM II/Incor) with		Concomitant aortic valve replacement and LVAD
(11)	Single institutional		-AV replacement	19	implantation is not associated with an impaired outcome
			-No AV procedure	299	
Rajagopal et al. (12)	Retrospective Single institutional	2013	LVAD (HM-XVE/Novacor/HM II/HVAD/Ventracor VentaAssist)	184	*De novo* or progression of native AI more pronounced with Cf-LVADs to control cohort (medical treatment)
			Control	132	
Cowger et al. (13)	Retrospective Single institutional	2014	LVAD (HM II)	166	Albeit common post LVAD implantation, AI was not seen to affect survival
Hiraoka et al. (14)	Retrospective Single institutional	2015	LVAD (HM II/HVAD/Ventracor VentaAssist)	99	AI was not seen to affect survival at 1 year
Robertson et al.	Retrospective	2015	LVAD (mostly HM II) with		AV closure was associated with increased mortality when
(15)	Registry (INTERMACS)		-AV closure	125	compared with repair or replacement in patients with AI
			-AV repair	95	who underwent LVAD implantation
			-AV replacement	85	
Holley et al. (16)	Retrospective Single institutional	2017	LVAD (HM II)	237	AI was seen to increase over time without having an impact on long-term mortality
Truby et al. (17)	Retrospective Registry (INTERMACS)	2018	LVAD (Continuous Flow LVADs)	10,603	1,399 patients on LVAD support developed moderate to severe AI; investigators showed negative impact on hemodynamics, hospitalizations, and survival
Tanaka et al. (18)	Retrospective	2020	LVAD (HM II/HVAD) with		Uncorrected mild aortic insufficiency had a higher risk of
	Single institutional		-mild AI	111	progression to moderate or greater aortic insufficiency
			-trace or no AI	493	after left ventricular assist device implantation with worse functional status and higher incidence of heart failure related readmission
Jimenez Contreras	Retrospective	2022	LVAD		A trend for less progression to moderate/severe AI seen
et al. (19)	Single institutional		-HM II	452	with HM3 implantation
			-HM 3	252	

AI, aortic insufficiency; AV, aortic valve; LVAD, left ventricular assist device; HM2, heartmate 2; HM3, heartmate 3; HVAD, heartware ventricular assist device.

Patients developing moderate to severe AI during follow-up exhibit significantly higher left ventricular end-diastolic diameter, reduced cardiac output, and higher levels of brain natriuretic peptide. Furthermore, reduced left ventricular unloading in this circumstance is ultimately reflected back toward the unsupported right ventricle, increasing right ventricular afterload. This predisposes to right ventricular failure and potentially limits the duration in which a single ventricular support configuration will be viable for the patient, which of course is paramount concern for the DT patient who is unlikely to have an alternative viable support strategy. With these thoughts in mind, it is unsurprising that significant AI after LVAD implantation has correlated with higher rates of rehospitalization and mortality conditional upon survival to 1 year (17). Another extremely rare but dreaded complication that may be seen in this clinical setting is aortic valve and aortic root thrombosis (20).

### Aortic insufficiency at index LVAD procedure

The decision of whether and/or how to intervene on pre-existing aortic insufficiency at the time of LVAD implant is influenced by a variety of factors. Chief among them is the severity of aortic pre-existing insufficiency. Traditionally, moderate or greater AI has prompted intervention while mild AI at the time of LVAD implant has often been managed without procedural intervention (6). However, as will be discussed in greater detail later on, it is now well appreciated that AI is likely to worsen with time following LVAD support. Therefore, particularly in the DT population where duration of LVAD support may be anticipated to be relatively longer in comparison to bridge to transplant patients, there may be consideration for correction of even mild degree of AI at the time of LVAD implant.

Once the decision to intervene on the aortic valve at the time of LVAD implant has been made, a variety of surgical approaches to deal with aortic valve pathology during LVAD implantation have been described including aortic valve closure (21, 22), repair (23, 24), and replacement (11, 25). In the presence of previous mechanical aortic valve replacement, closure techniques with a sandwich plug or patch (22, 26) have been described although most groups prefer converting these valves to bio-prostheses. With in situ bio-prostheses perioperative assessment will dictate the requirement of replacement if there is evident structural deterioration. In native aortic stenosis the degree of preexisting AI in the case of mixed disease will guide the need for intervention.

Robertson et al. (15) demonstrated that aortic valve closure was associated with increased mortality in comparison to aortic valve repair/replacement analyzing INTERMACS data from 305 patients who underwent concomitant aortic valve procedures during LVAD implantation; an increased incidence of postoperative AI was the pathophysiologic trade-off observed with aortic valve repair. The main concerns with aortic outflow tract closure are the potential catastrophic outcome in the setting of sudden pump failure as well as the limitations that will be encountered in the event of myocardial recovery and consideration of LVAD decommission. Many groups have implemented central oversewing to approximate the fibrous nodules of Arantius (Park's stitch) (27) to deal with preoperative AI with variable mid- and long-term outcomes in regards to AI recurrence (28–30). The decision-making in this paradigm is heavily influenced by an attempt to limit aortic cross-clamp time; operative experience, quality of aortic leaflet tissue, as well as projected time of support are all factors to be considered by the operating surgical team.

### Aortic insufficiency after LVAD implantation

Multiple studies have demonstrated that AI during continuous flow LVAD support is a progressive disease (9, 17). Reviewing INTERMACS data from 1,399 patients who developed moderate to severe AI during follow-up, Truby et al. (17) reported a temporal increase in the prevalence of significant AI with predictors of worsening AI including older age, female sex, smaller body mass index, mild pre-implantation AI, and DT. Recent data has shown the impact of uncorrected mild AI at the index implantation with 44% developing moderate or greater AI within 2 years follow-up (18); interestingly 9% of patients with no AI at the original implantation were seen to develop *de novo* AI. Failing conventional medical treatment strategies for AI, including blood pressure control (goal mean of 60–80 mmHg), diuretic therapy, and pump speed optimization with concomitant right heart catheterization (31), more definitive treatment will be required.

Conventional surgical approaches to ameliorate post-LVAD AI have been carried out with good results, accepting the risks of redo sternotomy and right ventricular injury as well as failure (32). In order to reduce procedural risk in this cohort of comorbid patients, percutaneous transcatheter approaches including transcatheter aortic valve replacement (TAVR) and percutaneous occlude devices of native or bioprosthetic prostheses have been developed (33–36). In a systematic review and meta-analysis of percutaneous transcatheter interventions for AI in continuous flow LVAD, TAVR and occlude devices demonstrated similar efficacy in significantly reducing severe AI (37).

Although variable device success has been demonstrated with TAVR for native pure AI (38), encouraging data has been produced from second-generation transcatheter heart valves that incorporate leaflet-clasping mechanisms to anchor themselves in the absence of valvular apparatus calcification (39). Such devices may become an important part of the armamentarium to address post-LVAD AI.

### Preventative measures

In a meta-analysis of eight studies with a total of 548 patients, Gasparovic et al. (40) reported a pooled incidence of *de novo* AI of 37%, with predictors of development and progression being older age, persistent aortic valve closure, female sex, and duration of LVAD support. Furthermore, Patil et al. (41) reported systolic blood pressure at 3 months, aortic valve closure and longer support duration being independent predictors of *de novo* AI following LVAD implantation. It is therefore pertinent that pump speed optimization takes place under hemodynamic and echocardiographic guidance prior to discharge, especially in patients fitting the above criteria. Strict blood pressure control during follow-up in combination with continuous outpatient hemodynamic and echocardiography-directed pump speed optimization allowing for at least intermittent AV opening is thought to potentially reduce the development and progression of AI after LVAD implant. By allowing intermittent aortic valve opening there is putatively less aortic commissural fusion and aortic root dilation, both of which are mechanisms for the development of AI post LVAD implant. Pulsatility or intermittent low-speed algorithms that may facilitate aortic valve opening may also prove of clinical significance in the future (42).

As commencement of LVAD support will instantly decrease left ventricular end-diastolic pressure and increase proximal ascending aortic pressure, the resulting increase in transvalvular gradient may unveil clinically significant AI that was “masked” by severe heart failure (15). This is probably even more applicable to patients with pre-existing increased proximal ascending aortic dimensions (43). Intraoperative assessment of the aortic valve pre- as well as post- LVAD implantation is therefore recommended in the context of DT.

Regarding intraoperative procedural modifications, the field of computational fluid dynamics (CFD) has offered a great degree of translational insight. Callington et al. (44) demonstrated that a lower outflow graft anastomosis location with appropriate angulation (inclination angle ≥90°, azimuthal angle of 60°or 120°) might reduce blood flow stagnation in the aortic root and produce normal wall shear stress and moderate pressure values in the region. Part of the authors' hypothesis was that a high root pressure due to the jet flow might contribute to *de novo* development of AI post LVAD implantation. Furthermore, an LVAD management strategy that allows intermittent AV opening has been shown with CFD simulations of blood flow, including platelet-surrogate dynamics, to improve biocompatibility by promoting platelet washout, reducing stasis, and decreasing thrombogenicity (45). More recently, Kasinpila et al. (46) also have shown that development of AI is associated with increased flow recirculation and turbulent eddies at the aortic root region; the distance from aortic root to the outflow graft was smaller in patients who developed AI.

## Mitral valve

Mitral regurgitation (MR) affects up to 10% of the general population, making it the most common heart valve disorder (47). In patients admitted with decompensated heart failure, between 36 and 53% of patients have MR of at least moderate severity, and its presence is associated with a poorer prognosis (48–50). The mitral valve and its apparatus forms a complex structure, and its function is intrinsically linked to left ventricular size and function. Amongst patients with heart failure the most common etiology is functional MR. Adverse ventricular remodeling leads to annular dilation and papillary muscle displacement, resulting in leaflet tethering and failure of coaptation. Impaired systolic function and ventricular dyssynchrony reduce the valve closing forces and further contribute to leaflet tethering. MR itself leads to increased volume loading of the left ventricle (LV), resulting in further LV dilation and creating a vicious cycle. MR may be secondary to other conditions, such as rheumatic heart disease or congenital abnormalities, and may be the primary cause of heart failure, or exacerbate cardiac insufficiency in a patient with co-existing heart failure. Gene expression analysis of myocardium from patients with significant MR undergoing left ventricular assist device (LVAD) implantation show increased expression of genes associated with inflammation, and reduced expression of cell energetics and proliferation genes, suggesting that these patients are a distinct subset of patients with cardiomyopathy, which may impact on response to therapies (51).

Conventional heart failure pharmacological treatments and cardiac resynchronization therapy have been shown to reduce the severity of MR through positive remodeling and reduction in the degree of ventricular dyssynchrony (52, 53). Prospective trials of percutaneous mitral valve edge to edge repair in patients with functional MR have provided mixed results. However, there may be benefit in a subset of patients with severe MR and LV systolic impairment on optimal medical therapy (54, 55). Functional MR can also be treated with conventional mitral valve repair or replacement, either alone or at the time of other surgical procedures such as coronary artery bypass grafting. Repair is associated with high rates of recurrent MR, and the benefits in terms of long term clinical outcomes has not been established (56). A prospective study of percutaneous mitral valve repair in patients listed for heart transplantation reported a procedural success rate of 87.5%, with low complication rates. Almost one quarter of patients were taken off the transplant list at 1 year due to clinical improvement, suggesting that this is viable therapy in patients with advanced heart failure (57). There have been concerns regarding the effect of percutaneous mitral valve repair on subsequent LVAD placement, as the functional mitral stenosis may affect left ventricular filling. However, a propensity matched study of 27 patients with prior percutaneous valve repair demonstrated similar 2-year outcomes to a matched group with untreated functional MR with pulmonary artery and wedge pressures being lower in patients with prior valve repair (58).

In patients with end stage heart failure that has proven refractory to conventional heart failure therapies, approximately one third have at least moderate to severe MR (59). Effective LVAD therapy leads to mechanical unloading of the LV and a reduction in pulmonary artery pressures. This leads to changes at the myocyte and biochemical level, resulting in positive ventricular remodeling, and reduction in left ventricular volumes (60, 61). The marked early improvement in MR severity in most patients following LVAD implantation alone means that concomitant mitral valve surgery is rarely required. In the pivotal MOMENTUM 3 study, which compared a third generation centrifugal LVAD, the HeartMate 3 (Abbott, Abbott Park, IL), with a second-generation axial flow pump, the HeartMate II, 43.5% of patients had at least moderate MR or greater prior to implantation and did not undergo concomitant mitral valve intervention (62). At 1 month following implantation, 6.2% of patients treated with the HeartMate 3 device had residual MR, as compared to 14.3% in the HeartMate II arm. After 2 years of LVAD support, the proportion of patients with clinically significant MR remained low, 9.4% in the HeartMate 3 group vs 15.4% in the HeartMate II arm.

In an INTERMACS analysis that examined all LVAD implantations between 2008 and 2014, 263/4930 adults with moderate to severe MR underwent a concomitant mitral valve procedure, of whom 96% received a mitral valve repair (63). Patients undergoing mitral valve intervention had higher pulmonary artery pressures, more severe MR, and were more likely to have had prior mitral valve intervention. No difference in short- or long-term survival was seen in patients undergoing mitral valve procedures, although there was a reduction in re-hospitalization, predominantly due to a reduction in right heart failure. A systematic review of 8 studies examining the role of mitral valve intervention at the time of LVAD implantation failed to show a survival benefit as compared to LVAD implant alone (64). Consensus guidelines supported by the ISHLT and American Association for Thoracic Surgery state that routine repair or replacement for severe MR is not recommended. Routine replacement of a properly functioning mechanical mitral valve is also not recommended.

Data on long term outcomes in patients with residual MR is conflicting. A recent INTERMACS analysis of patients receiving implants between 2006 and 2017 revealed that 18.8% of patients had at least moderate MR at 3 months post LVAD implant (65). Incidence of late right heart failure and renal failure were higher post-operatively, and there was a trend toward increased longer term mortality. Similar findings were also seen in a single center, which revealed that in the 20% of patients with residual MR, right ventricular function was worse and dimensions larger. Time to first hospitalization was significantly shorter amongst those with significant MR (66). However a more contemporary analysis incorporating data from the MOMENTUM 3 study and continued access protocol showed no difference in survival, rehospitalization rates or incidence of right heart failure in patients with residual MR (67).

There is limited data examining the impact of residual MR specifically in patients receiving an LVAD as DT. In one study that included 91 patients, 68% had moderate or severe MR. The presence of at least moderate MR was an independent predictor of reduced survival at 30 days and 2 years (68). In the previously discussed INTERMACS analysis of concomitant mitral valve procedures, there was no overall benefit from intervention. However, in the subgroup of patients implanted as an initial DT strategy, there was a trend for higher 2-year survival for patients that underwent mitral valve intervention (73% vs 64%, *p* = 0.09) (63). This data would suggest a potential benefit of mitral valve intervention in the subset of LVAD patients implanted as DT, although the numbers analyzed are too small to draw definitive conclusions.

Predicting which patients are likely to be left with residual MR is challenging. Those at increased risk appear to be younger, more likely to be female, non-Caucasian, with non-ischemic etiology of heart failure (65). They also typically have worse right ventricular function, more tricuspid regurgitation (TR) and higher pulmonary artery pressures (66). More severe MR at baseline, and larger LV end diastolic diameter are consistent risk factors across different cohorts (69). A single center study identified that patients with persistent atrial fibrillation and larger left atrial dimensions were less likely to achieve a significant reduction in MR severity, and had worse long term survival (70). This suggests that LVAD therapy is less effective at left atrial remodeling and may have limited impact on MR severity if left atrium enlargement is a significant contributor to mitral annular dilation. Posterior displacement of the mitral coaptation point also predicts residual MR risk (71). While those with predominantly Carpentier type 1 MR due to annular dilation are likely to improve following LVAD implantation, type IIIb valve dysfunction due leaflet and chordae restriction may be less likely to improve, as LVAD unloading will reduce closing forces and may further limit coaptation (72).

Perioperative measures may reduce the risk of residual MR. Appropriate inflow cannula alignment, as determined by a combined assessment of anterior and lateral angulation was associated with greater improvement in MR severity at 1 month (73). Use of centrifugal flow LVAD pumps is also associated with a greater reduction in MR, as compared to axial flow pumps (62). Hemodynamic optimization of LVADs is a key component of long-term care. Selection of the most appropriate pump speed through ramp testing and right heart catheterization have been shown to reduce pulmonary capillary wedge pressures, through improved mechanical unloading (74). Whether this translates to a reduction in MR severity has not been assessed. Institution of guideline directed heart failure therapies in patients with long term LVADs has been shown to improve survival and quality of life (75). One small prospective study demonstrated that medical therapies in LVAD supported patients aids remodeling through a reduction in left ventricular dimensions and mass more than LVAD alone, however there was no impact on the degree of MR (76). A comprehensive list of representative studies regarding the interplay of MR during LVAD implantation is provided in Table 2.

**Table 2:** Key studies in chronological order of publication reporting on the interaction of MR and LVAD implantation.

**References**	**Design**	**Year**	**Study groups**	***N***	**Main outcome**
Taghavi et al. (77)	Retrospective Multi-institutional	2013	LVAD (HM II) with -MV intervention -No MV intervention	21 36	No difference in survival at 1 year. MV intervention was associated with a decrease in pulmonary vascular resistance
Goodwin et al. (78)	Retrospective Single institutional	2017	LVAD (HM II/HVAD) with <moderate-severe MR ≥ moderate-severe MR	195 43	Resolution of MR was sustained at 180 days post LVAD implantation. No difference in survival was seen between two groups
Kassis et al. (66)	Retrospective Single institutional	2017	LVAD (CfLVADs)	69	Significant residual MR post-LVAD implantation was associated with persistent pulmonary hypertension, worse RV function, and significantly shorter time to hospitalization and death
Fukuhara et al. (79)	Retrospective Single institutional	2017	LVAD (HM II/HVAD/VentracorVentaAssist/ DuraHeart/DeBakey VAD) with >moderate MR and -MV repair -no MV repair	52 63	Concomitant MV repair was associated with less frequent late right heart failure
Dobrovie et al. (80)	Retrospective Single institutional	2018	LVAD (HM II/HVAD) with None to moderate MR Severe MR	63 65	Preoperative severe MR resolves in most patients early on after LVAD implantation and is not associated with worse clinical outcomes or intermediate-term survival
Robertson et al. (63)	Retrospective Registry (INTERMACS)	2018	LVAD (LVADs) and -MV repair -MV replacement -No MV procedures	252 11 4,667	Concomitant MV procedure was not shown to improve survival, but a trend toward increased survival was seen in DT patients with moderate to severe MR who underwent MV procedure
Kawabori et al. (81)	Retrospective Single institutional	2019	LVAD (HM II/HVAD) with severe MR and -MV procedure -no MV procedure	26 82	Investigators did not identify any advantage in outcomes for patients who underwent MV procedure
Okoh et al. (68)	Retrospective Single institutional	2019	DT LVAD (HM II) with baseline MR <moderate MR ≥ moderate MR	29 62	≥ moderate MR was seen to be associated with worse survival at both short and midterm follow-up
Pawale et al. (82)	Retrospective Single institutional	2019	LVAD (HM II/HM 3/HVAD) with severe MR and -MV procedure -no MV procedure	78 28	Concomitant MV repair can be carried out safely during LVAD implantation. Investigators suggest a better reduction in MR severity and reduced rate of readmission for heart failure
Kanwar et al. (62)	Retrospective Registry (MOMENTUM 3 trial)	2020	LVAD with >moderate MR -HM II -HM 3	206 197	HeartMate 3 was seen to improve clinically significant MR earlier, sustainably, and to a greater degree than HeartMate 2. Outcomes following LVAD implantation were not influenced by baseline or residual MR
Cruz Rodriguez et al. (83)	Retrospective Single institutional	2021	LVAD (HM II/HVAD)	111	Residual moderate to severe MR was found to be present in 1/4 of patients. An association was found with increased incidence of right heart failure, higher mean pulmonary pressure, and pulmonary capillary wedge pressure with no effect on 1 year survival
Jain et al. (65)	Retrospective Registry (INTERMACS)	2022	LVAD (CfLVADs)	8,364	18.8% of patients were found to have residual MR with concomitant mitral valve procedures appear to reduce this risk. Residual MR was associated with worse clinical outcomes

MR, mitral regurgitation; MV, mitral valve; DT, destination therapy; LVAD, left ventricular assist device; HM2, heartmate 2; HM3, heartmate 3, HVAD, heartware ventricular assist device.

### Mitral stenosis and prosthetic mitral valves

Mitral stenosis impairs left ventricular filling that leads to reduced flows in an LVAD supported patient. Therefore, mitral valve repair or replacement is recommended in patients with moderate or severe mitral stenosis of any cause. The presence of a prosthetic mitral valve is not a contraindication to LVAD implantation. Trans mitral flow typically improves following LVAD implantation, therefore the risk of thrombus formation is low. The 2019 European Association of Cardiothoracic Surgeons Expert Consensus recommend that ‘Exchange of a functional mitral mechanical or biological prosthesis at the time of long-term mechanical circulatory support device implantation is not recommended (84).

## Tricuspid valve

Moderate or severe tricuspid regurgitation (TR) is seen in around 20% of patients with chronic heart failure, and around a third of patients presenting with acute heart failure (85). Its prevalence increases as heart failure severity worsens and is associated with higher morbidity and mortality (86).

Right ventricular remodeling is a common consequence of left ventricular systolic impairment and/or left sided valve dysfunction, because of pulmonary arterial hypertension. This causes tricuspid annular dilation and leaflet tethering, leading to functional TR. High right ventricular preload due to venous congestion also leads to volume loading of the right ventricle, increasing the degree of TR. A significant proportion of patients with chronic heart failure have cardiac implantable electronic devices and leads crossing into the right ventricle can also impair tricuspid valve closing. The right ventricle is sensitive to volume loading conditions, relief of venous congestion through effective diuresis can lead to favorable right ventricular remodeling and reduce the degree of TR (87). Targeted pulmonary vasodilator therapies in patients with left sided heart failure have not shown to be of significant clinic benefit and may be harmful (88).

Right ventricular failure remains a common early complication following LVAD implantation, and is associated with prolonged intensive care stays and increased mortality (89). Right heart failure following LVAD arises from a multitude of factors. Higher left sided output provided by the LVAD increases the preload delivered to a deconditioned right ventricle. Furthermore, displacement of the interventricular septum to the left side alters RV geometry and may further exacerbate TR. Perioperative transfusion of blood products, and hypoxia can place additional stress on the right ventricle. Nevertheless, predicting which patients will develop right heart failure remains a challenge, and requires a multi-modality assessment, combining clinical factors, cardiac imaging, and hemodynamic assessment. Severe TR was shown to be an independent risk factor for the requirement of mechanical right ventricular support in one study and was incorporated into a risk scoring system (90). However, larger retrospective analyses have failed to show that TR severity is an independent marker of risk for right heart failure (91–93).

TR typically improves in the first month following LVAD implantation, as the reduction in pulmonary artery pressures aids right ventricular remodeling. A EUROMACS registry study demonstrated that 65% of patients with moderate to severe TR pre implant have no to mild TR at 30 days post-implant (94). Patients with idiopathic dilated cardiomyopathies were more likely to improve as compared to other etiologies.

Despite the natural improvement in tricuspid valve competence post-LVAD in the short-medium term, the presence of at least moderate TR appears to complicate the early post-operative course, with a higher need for mechanical right ventricular support, prolonged inotrope use and intensive care stay (95). Whether TR is itself the cause, or whether it is simply a marker of severity of pre-operative right ventricular dysfunction remains debatable. Surgical correction of TR increases right ventricular afterload, which in turn may further compromise the function of a deconditioned right ventricle.

Concerns regarding early right ventricular recovery likely explains why tricuspid valve repair is the most frequent concomitant valve intervention performed at the time of LVAD implantation (96). However, there is wide variability in practice amongst different centers, with around one quarter of patients with moderate to severe TR undergoing tricuspid valve procedures, most commonly tricuspid annuloplasty (97).

Single center retrospective studies have suggested a reduction in rates of early right heart failure, improved postoperative outcomes, and reduced early rehospitalization in patients undergoing concomitant TV repair, without a clear survival benefit (98–100). A systematic review of eight retrospective studies showed no difference in rates of right heart failure, renal failure, early or late mortality (101). However, the group undergoing tricuspid intervention were sicker at baseline, with higher bilirubin levels and central venous pressure, which commonly portend a poorer prognosis. As these patients had similar post-operative outcomes, the authors suggested that tricuspid valve intervention may ameliorate this excess risk. Tricuspid valve intervention increased cardiopulmonary bypass time by an average of 35 min in this meta-analysis.

Larger registry database analyses have consistently failed to show a benefit from concomitant tricuspid valve intervention. A stratified INTERMACS registry analysis of 8,263 patients revealed an increased risk of adverse events, including bleeding, arrhythmia and stroke, and higher mortality in patients with moderate to severe TR undergoing valve intervention (102). Similarly, a Society of Thoracic Surgeons database analysis revealed an excess of adverse events in patients with significant TR undergoing concomitant tricuspid valve intervention, including higher rates of renal dysfunction, reoperation, and blood transfusion, as well as prolonged intensive care stay (103).

A prospective randomized controlled trial of tricuspid valve intervention (annuloplasty or replacement) vs no intervention was recently presented at the 2022 American Association of Thoracic Surgeons meeting (TVVAD trial) (104). The primary endpoint was incidence of right heart failure at 6 months. The trial was stopped early due to futility after enrolment of 60 patients. No differences were seen in any of the secondary endpoints, including all-cause mortality.

The durability of tricuspid valve repair at the time of LVAD implant is questionable, with between 21 and 37.8% of patients developing at least moderate TR at follow up (97, 105, 106). This was associated with higher rates of late right heart failure.

The European Association of Cardiothoracic Surgeons Expert Consensus on long term mechanical circulatory support recommend “Re-evaluation of patients with moderate to severe TR after treatment with diuretic therapy, if condition permits” (class 1C) and “In carefully selected patients, tricuspid valve repair for moderate to severe TR at the time of long-term mechanical circulatory support implantation may be considered” (Class IIb C) (84). This consensus document was published prior to the large INTERMACS analysis described earlier and the recently concluded TVVAD randomized trial. Furthermore, no studies have identified a specific subgroup of patients who may benefit from a concomitant TV procedure. Therefore, it is difficult to know which parameters to use in clinical decision making when selecting patients for concurrent TV repair, if this should be done at all. Future prospective studies should assess the impact of baseline factors, such as hemodynamic measures of right ventricular performance, echocardiographic measures such as TV annular diameter, TR severity (moderate vs severe), and clinical factors including INTERMACS status and inotrope score, to develop a personalized approach to assessing need for concomitant tricuspid valve intervention. A comprehensive list of representative studies regarding the interplay of TR during LVAD implantation is provided in Table 3.

**Table 3:** Key studies in chronological order of publication reporting on the interaction of TR and LVAD implantation.

**References**	**Design**	**Year**	**Study groups**	***N***	**Main outcome**
Piacentino et al. (99)	Retrospective Single institutional	2011	LVAD with severe TR -TV procedure -no TV procedure	34 81	Concomitant TV procedure was associated with improved early clinical outcomes. Furthermore, a trend toward improved overall survival was documented for the TV procedure cohort
Robertson et al. (103)	Retrospective Registry (STS Database)	2014	LVAD (CfLVADs) with >moderate TR -TV procedure -no TV procedure	588 1,608	Concomitant TV procedure during LVAD implantation for moderate to severe TR did not reduce early death or right VAD requirement. Investigators documented overall worse early postoperative outcomes
Song et al. (97)	Retrospective Registry (INTERMACS)	2016	LVAD (CfLVADs as DT) with >moderate TR -TV procedure -no TV procedure	215 757	Concomitant TV procedure did not result in improved survival with 21%−27% of patients undergoing TV procedure developing recurrent late TR
Critsinelis et al. (100)	Retrospective Single institutional	2018	LVAD (HM II/HVAD) with severe TR	59	Concomitant TV procedure did not impact patient outcomes but did reduce the incidents of 30-day readmission
Barac et al. (105)	Retrospective Single institutional	2020	LVAD (Durable) and TV procedure	156	37.8% of patients undergoing TV ring annuloplasty at the time of LVAD implantation had recurrent TR at intermediate follow-up. This was independently associated with late right heart failure
Veen et al. (94)	Retrospective Registry (EUROMACS)	2021	LVAD (uncorrected TR) -mild to moderate -moderate to severe	1,690 806	Uncorrected TR was associated with increased early as well as late mortality. On average TR was seen to diminish progressively following LVAD implantation. Investigators suggested TR grade should not be the sole criterion for patient selection for TV procedure

TR, tricuspid regurgitation; TV, tricuspid valve; LVAD, left ventricular assist device: HM2, heartmate 2; HM3, heartmate 3; HVAD, heartware ventricular assist device.

## Multiple valve pathology

Some degree of pathology involving multiple valves is commonly encountered in the advanced heart failure population undergoing LVAD implant. When there is significant pathology involving multiple valves, concomitant multiple valve intervention with LVAD implant may need to be considered. There is limited empirical data to guide clinicians in prognostication around LVAD implant with multiple concomitant valve intervention (summarized in Table 4), but it is intuitive that the longer cross-clamp and cardiopulmonary bypass that are associated with multiple valve intervention may predispose the patient to greater risk at that the time of LVAD implant. In general, the principles outlined above for individual valve pathologies may form a starting point decision making, but clearly the ultimate the decision over whether to move forward with LVAD implant surgical plan will need to be individualized based on patient-specific factors when confronting concomitant multiple valve pathology. Nevertheless, contemporary data does suggest that LVAD implant with concomitant multiple valve intervention can be undertaken with acceptable outcomes (107–110). In a single-center experience of concomitant valve procedures during LVAD implantation, Sugiura et al. (109) elegantly demonstrated no association with mortality; investigators reported on a cohort of 91 patients including 29 double valve procedures. Patients undergoing concurrent valve procedures did, however, have significantly higher risk of right heart failure as well as stroke that may be partially attributed to the longer cardiopulmonary bypass and cross-clamp times. The largest to date cohort of patients undergoing multiple valve procedures during LAVD implantation stems from analysis of the MOMENTUM 3 trial, which included 85 patients who underwent multiple valve intervention (110). An adjusted analysis performed by the investigators did not identify any difference in survival at 1 or 2 years between single and multiple valve procedures. These findings suggest that in selected patients undergoing LVAD implant concomitant valve intervention does not pose prohibitive risk.

**Table 4:** Key studies in chronological order of publication reporting on the outcomes of concurrent multiple valve procedures during LVAD implantation.

**References**	**Design**	**Year**	**Study groups**	***N***	**Main outcome**
John et al. (107)	Retrospective Multi-institutional	2014	LVAD (HM II) -No valve procedure -Single valve procedure -Multiple valve procedure	641 205 37	Multiple valve procedures were associated with a higher 30-day (*p* = 0.04), as well as 2-year (*p* = 0.046) mortality
Maltais et al. (108)	Retrospective Multi-institutional	2016	LVAD (HM II/HVAD) -No valve procedure -Single valve procedure -Multiple valve procedure	398 190 26	Investigators concluded that survival was comparable between groups with no influence from concomitant procedures
Sugiura et al. (109)	Retrospective Single institutional	2019	LVAD (HM II/HVAD) -No valve procedure -Single valve procedure -Multiple valve procedure	435 62 29	Concomitant procedures were not associated with increased mortality
John et al. (110)	Retrospective Registry (MOMENTUM 3)	2022	LVAD (HM3) -No valve procedure -Single valve procedure -Multiple valve procedure	1,380 325 85	Adjusted analysis did not identify any difference in survival at 2 years between single and multiple valve procedures

LVAD, left ventricular assist device: HM2, heartmate 2; HM3, heartmate 3; HVAD, heartware ventricular assist device.

## Discussion

There appears to be a signal for temporal reduction in mortality risk associated with concomitant valve procedures during index LVAD implantation. Earlier studies have reported 30-day mortality rates ~25% with combined aortic valve procedures, a nearly 5-fold increase relative to isolated implants (111). In contrast, in the more contemporary landscape, there is a growing body of literature that supports that concomitant valve surgery during LVAD implantation can be delivered without impacting survival, in selected patients (98, 108, 112). Although LVAD clinicians' knowledge and ability to manage concomitant valve disease in LVAD patients has likely improved over time, it is also true that LVAD technology has significantly evolved. This becomes evident from published outcomes of the Multicenter Study of MagLev Technology in Patients Undergoing Mechanical Circulatory Support Therapy with HeartMate 3 (MOMENTUM 3), a multicenter, 1:1 randomized, pivotal study comparing the treatment efficacy of the HeartMate 3 LVAD with the HeartMate II LVAD in patients with advanced-stage HF (62, 113). The HeartMate 3 was shown to be more efficient at hemodynamic unloading of clinically significant MR early, sustainably, and to a greater extent. Furthermore, uncorrected baseline as well as residual MR had no influence on outcomes after LVAD implantation at 2-year follow-up (62). Further data from the MOMENTUM 3 trial portfolio that includes 2,200 patients, investigated the outcomes of 466 patients who underwent a concomitant valve procedure at the index LVAD implantation (110). Carrying out concomitant valve procedures exposed patients to early postoperative morbidity including stroke, bleeding, and right heart failure, but there was no detectable difference in 30-day mortality and 2-year survival. Furthermore, no difference in outcomes amongst patients with significant mitral or tricuspid regurgitation irrespective of corrective surgery was demonstrated. Based on their findings, investigators suggested that sufficient equipoise exists to consider a randomized trial assessing the benefit of commonly performed valve interventions during LVAD implantation. In conjunction with growing transcatheter-based procedural arsenal for the amelioration of valve disease, findings such as these imply decisions and strategies around the management of concomitant valve pathology are likely going to continue to evolve as the field moves forward.

The impact of valvular pathology on outcome measures in critically ill patients is well described (114). “Surgical acumen” instinctively dictates anatomical correction of all cardiac pathology to allow for best chance of myocardial recovery or remodeling. Despite many institutional as well as registry studies describing improved outcomes for some valvular pathology correction during LVAD implantation, the jury remains out for several individual pathologies. The question that comes to mind is: are patients being exposed to higher risk implantations with increased perioperative morbidity and no detectable difference in outcomes? Individualized evidence-based medicine may answer partly this question; for example, smaller females undergoing LVAD implantation with pre-existing mild AI is probably a cohort that requires concomitant aortic valve intervention (19). As the majority of the relevant studies do not report long-term outcomes that are particularly pertinent in DT implant population, the necessity for additional prospective and longer-term follow up studies is evident. In the meantime, management strategies that include maintaining euvolemia, blood pressure control, and optimized hemodynamics allowing for intermittent aortic valve opening are imperative at reducing complications.

## Author contributions

ID, PC, and JK performed literature review, manuscript preparation, and critical revision. MK, JP, CB, and CM performed critical revision. All authors approved of the final version of the manuscript prior to submission.

## Conflict of interest

The authors declare that the research was conducted in the absence of any commercial or financial relationships that could be construed as a potential conflict of interest.

## Publisher's note

All claims expressed in this article are solely those of the authors and do not necessarily represent those of their affiliated organizations, or those of the publisher, the editors and the reviewers. Any product that may be evaluated in this article, or claim that may be made by its manufacturer, is not guaranteed or endorsed by the publisher.
